# Circular inference in bistable perception

**DOI:** 10.1167/jov.20.4.12

**Published:** 2020-04-21

**Authors:** Pantelis Leptourgos, Charles-Edouard Notredame, Marion Eck, Renaud Jardri, Sophie Denève

**Affiliations:** 1 Laboratoire de Neurosciences Cognitives & Computationnelles, ENS, INSERM U960, PSL Research University, Paris, France; 2 Department of Psychiatry, Yale University, New Haven, CT, USA; 3 Univ. Lille, INSERM (U1172), CHU Lille, Lille Neuroscience & Cognition Research Centre (LiNC), PSY team, CURE platform, Fontan Hospital, Lille, France

**Keywords:** bistability, Necker cube, Bayesian inference, circular inference

## Abstract

When facing ambiguous images, the brain switches between mutually exclusive interpretations, a phenomenon known as bistable perception. Despite years of research, a consensus on whether bistability is driven primarily by bottom-up or top-down mechanisms has not been achieved. Here, we adopted a Bayesian approach to reconcile these two theories. Fifty-five healthy participants were exposed to an adaptation of the Necker cube paradigm, in which we manipulated sensory evidence and prior knowledge. Manipulations of both sensory evidence and priors significantly affected the way participants perceived the Necker cube. However, we observed an interaction between the effect of the cue and the effect of the instructions, a finding that is incompatible with Bayes-optimal integration. In contrast, the data were well predicted by a circular inference model. In this model, ambiguous sensory evidence is systematically biased in the direction of current expectations, ultimately resulting in a bistable percept.

## Introduction

Perception has been defined as the process of combining available information to create valid and useful interpretations of the world. Although our phenomenological experience prompts us to presume that perceptual decisions are trivial, the truth might be very different. An interesting example is the visual perception of depth. When we see an object, our brain must reconstruct its three-dimensional (3D) shape from a two-dimensional (2D) retinal image; in other words, the brain must solve an inference problem (Von Helmholtz, 1866). Unfortunately, these problems are ill-posed, as in most cases, the 2D retinal projection is compatible with many different 3D objects (Kersten, Mamassian, & Yuille, 2004). The brain must combine ambiguous information received by peripheral sensors (e.g., disparity cues, movement cues) with pre-existing information (either hard-wired or learned) concerning properties of the environment or the potential cost of a wrong decision to cope with perceptual uncertainty (Mamassian & Landy, 1998; Zhang, Xu, Jiang, & Wang, 2017). These combinations are expressed in Bayes’ theorem, in which prior probability distributions and sensory likelihoods are multiplied, resulting in a posterior probability distribution over possible solutions to the perceptual problem. Generally, only a single dominant (most probable) interpretation will emerge from these constraints.

**Figure 1.**
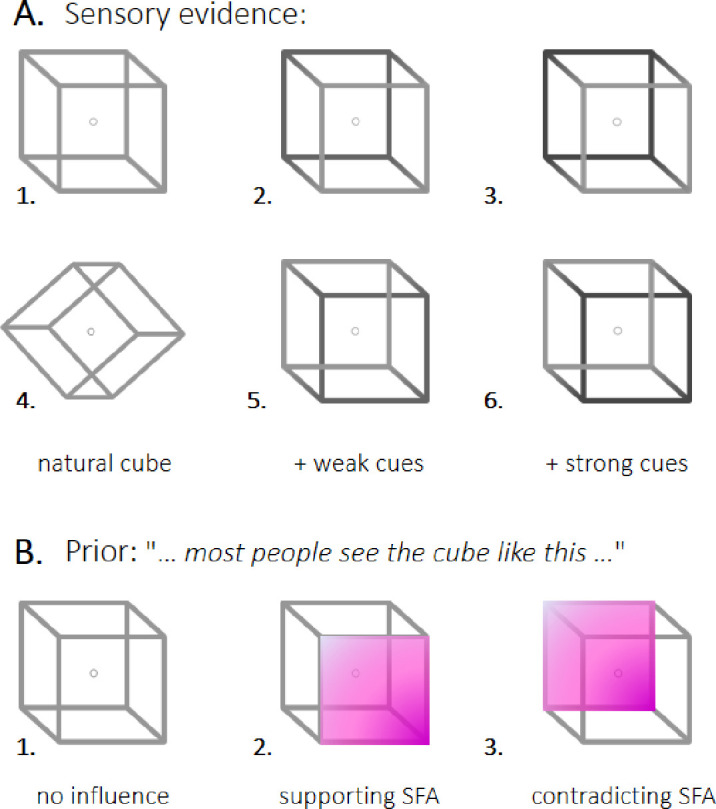
Stimuli and instructions. (A) Different Necker cubes were used to induce bistable perception, in which the 2D figure is perceived as a 3D cube with either the left or the right side located closer to the observer. Even in the case of the completely ambiguous stimulus (1), people have an implicit preference to interpret the cube as seen from above (SFA interpretation), which was interpreted as an implicit prior. This prior was refuted by tilting the stimulus (4). Sensory evidence was manipulated by adding visual cues in the form of contrasts (2-3 and 5-6). The contrast was strong (3 and 6) or weak (2 and 5) and supported (2 and 3) or contradicted (5 and 6) the implicit prior. (B) A further manipulation of the prior was achieved by providing correct or wrong information to the participants about which interpretation was generally stronger (explicit prior). The instructions either supported or contradicted the implicit prior. An additional control group received no particular instructions. Crucially, all groups received the same visual instructions (including the stimulus and the two possible interpretations) and the differences were only the verbal instructions to avoid additional priming effects. Note that the color used in the present figure has only been added for illustration purposes; during the experiment, participants were presented with full cubes.

However, when the level of ambiguity is too high, the identification of a single interpretation is not possible. Strikingly, ambiguous figures that are compatible with more than one plausible interpretation (Necker, 1832; Wheatstone, 1838) lead to *bistable* (or more generally *multistable*) perception (Blake & Logothetis, 2002). When presented with those figures, the perceptual system is unable to commit to a single stable interpretation and instead oscillates between mutually exclusive interpretations every few seconds. A famous figure known to induce bistability is the *Necker cube* (NC) (Necker, 1832) (Figure 1A), in which a 2D collection of lines is automatically interpreted as a 3D cube, which is either “seen from above” (SFA interpretation) or “seen from below” (SFB interpretation). Interestingly, the NC is an asymmetrical stimulus, as it generates an implicit preference for the SFA interpretation (i.e., the general preference of humans to interpret things as if they were located below the level of their eyes) (Dobbins & Grossmann, 2010; Mamassian & Landy, 1998).

Although the concept of perception as inference under uncertainty offers a principled method to explain the efficiency of perceptual systems and certain perceptual illusions, it less directly accounts for bistable perception. Indeed, if the brain uses explicit representations of uncertainty (e.g., a probability distribution instead of a point estimate) (Lochmann & Deneve, 2011; Ma, 2012; Ma & Jazayeri, 2014; Pouget, Dayan, & Zemel, 2003), ambiguous stimuli should be recognized as such and not generate a unique, persistent representation. Notably, bistable perception is far from unique in that case. Although many studies have reported that the brain is able to reach Bayes-optimal decisions (Ernstme & Banks, 2002; Körding et al., 2007; Shen & Ma, 2016; Weiss, Simoncelli, & Adelson, 2002), numerous tasks exist in which human behavior deviates significantly from a Bayesian observer (Acerbi, Vijayakumar, & Wolpert, 2014; Beck, Ma, Pitkow, Latham, & Pouget, 2012; Drugowitsch, Wyart, Devauchelle, & Koechlin, 2016; Hudson, Maloney, & Landy, 2007).

Deviations from Bayesian optimality might be the consequence of highly non-linear and state-dependent interactions between feedback and feedforward streams of information in brain circuits (Heeger, 2017). Some of these effects are quantified by the *circular inference* framework (Jardri & Denève, 2013). According to this framework, hierarchical processing in the brain is analogous to the propagation of probabilistic messages (beliefs) in a hierarchical model of the world (Bishop, 2006). The combination of feedforward and feedback inputs is equivalent to the product of a prior and likelihood in Bayes’ theorem. However, because neural circuits are highly recurrent, sensory evidence and prior information easily reverberate and are artificially amplified through feedforward/feedback loops in the brain, resulting in the corruption of sensory evidence by prior information and *vice versa*. This reverberation can be avoided if excitation (E) and inhibition (I) are perfectly balanced in cortical circuits (Jardri & Denève, 2013), a well-known property of the healthy brain (Okun & Lampl, 2008; Xue, Atallah, & Scanziani, 2014).

Recently, our team hypothesized a link between an E/I imbalance in patients with schizophrenia and the occurrence of psychotic symptoms (hallucinations and delusions). This hypothesis was recently reinforced by experimental evidence in a probabilistic reasoning task (Jardri, Duverne, Litvinova, & Denève, 2017). Interestingly, we also detected a certain amount of circularity in healthy participants, particularly the corruption of sensory evidence by prior information. If circular inference is a more general mechanism than initially predicted, an interesting question arises: is it possible to detect evidence of circularity (Leptourgos, Denève, & Jardri, 2017) in the perceptual behaviors of healthy subjects in the absence of any psychotic experience? Here, we propose that bistability represents an example of percepts induced by this type of circularity.

We induced bistability in healthy participants using the NC to investigate this theory. We asked how different pieces of information, including pre-existing priors (i.e., the SFA preference), newly acquired priors (i.e., instructions), and visual cues, were combined to generate the percept. We compared different Bayesian and circular inference (CI) models for their abilities to fit the data. We particularly sought to understand whether circularity and aberrant correlations between priors and sensory evidence significantly contributed to the way we perceive the world.

## Methods

This study adhered to the tenets of the Declaration of Helsinki. Participants were healthy volunteers meeting the following inclusion criteria: age > 18 years, provision of informed consent, normal or corrected-to-normal near visual acuity, no past or current medical history of neurological or psychiatric disorders, and no current or recent use of psychotropic medication or toxic drugs. Near visual acuity was quantified using the Parinaud score; we considered values ≤2 as normal. Of the 65 participants initially recruited, 10 were excluded because of outlying mean relative predominance values (with cutoffs set to *Q*1  −  1.5  ×  *IQR* and *Q*3  +  1.5  ×  *IQR*, where *Q*1 *and* *Q*3 are the lower and upper quartiles, respectively, and *IQR* is the interquartile range). Importantly, 7 of the 10 excluded participants also exhibited qualitatively bizarre behavior (such as opposite effects of visual cues (negative slopes), no effect of visual cues (flat curves with the relative predominance [RP] constantly at or below chance) or extreme values (close to 0 or 1) of the RP (particularly in the ambiguous or weak-cue condition)), indicating a misunderstanding of the instructions, low attention levels or fatigue (the exclusion of only those seven participants did not change any of the results; see Supplementary Figure S6).

### Experimental setting and procedure

**Figure 2.**
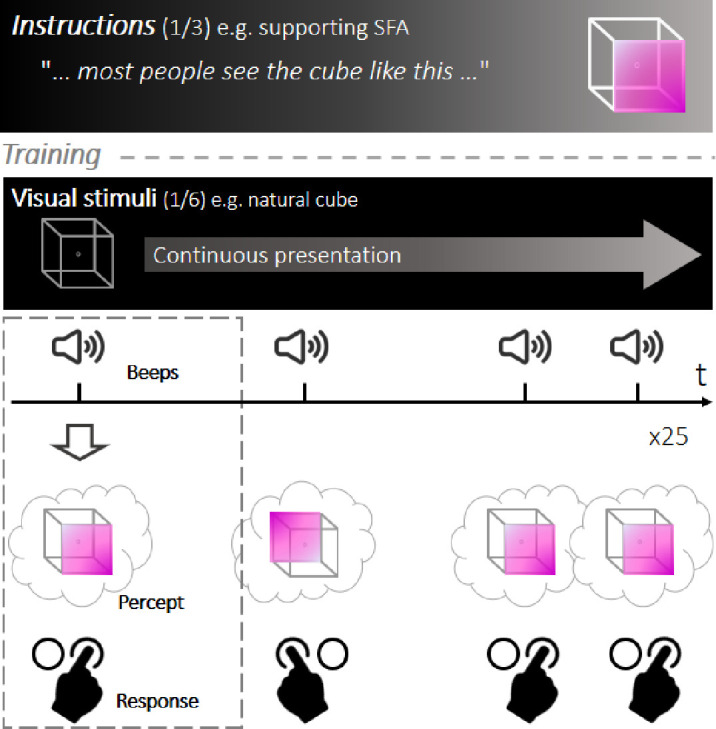
Experimental design. The task was inspired by a previous study (Mamassian & Goutcher, 2005). Instructions were provided at the beginning of the experiment (each participant received one set of instructions, creating a between-subjects design) and were followed by a short training phase to familiarize participants with the stimulus and the switches. During each run, one version of the cube was continuously presented to the participants, who were asked to discontinuously report their dominant percept by pressing a button every time a sound was heard. Each run consisted of 25 sound trials (mean inter-sound interval = 1.5 seconds). The main experiment consisted of 30 runs separated into six blocks of five runs each. In each block, a different variant of the stimulus was used. The first and fourth blocks always contained the ambiguous cube. The four cue conditions were randomly assigned to the four remaining blocks.

The general procedure (Figure 2) was inspired by the protocol devised by Mamassian and Goutcher (Mamassian & Goutcher, 2005) and consisted of six blocks of five consecutive runs. During each run, a 200 × 200 pixel NC displayed in the middle of a black screen was continuously presented to the participants. Using a forced-choice method, we asked participants to report their ongoing interpretation as soon as they heard a warning sound, which occurred 25 times in a pseudo-regular manner (mean inter-sound interval = 1.5 seconds, uniformly distributed between 1 and 2 seconds). Each response corresponded to a trial, providing a discontinuous sampling of the perceptual dynamics of the task. Runs were separated by a black screen that was presented for a duration of 10 seconds to minimize between-run influences. The experiment was also interspersed with five between-block breaks of non-predefined durations. Before the experiment, participants were informed that they would be presented with empty cubes, the two possible interpretations of which were explicitly described. The basic instruction was to passively view these cubes without trying to constrain perception.

We manipulated sensory evidence either by making the cubes homogeneously gray (i.e., perfectly ambiguous) or cuing them by shadows (Figure 1A (1-3 and 5-6)). This additional depth information was intended to bias perception toward one interpretation or the other. It was specified by two parameters. First, its orientation was defined in relation to the implicit prior. A shadow falling on the top left corner was expected to emphasize the SFA preference and thus was classified as a supporting cue. Conversely, a shadow that fell on the bottom right corner was characterized as a contradictory cue, as it differed from the implicit bias. Second, the strength of the cue (which is also potentially conceived in terms of the amount of sensory information) was controlled by the shadowing contrast level. Weak and strong cues corresponded to 20% and 30% contrast, respectively. The first and fourth blocks always consisted of the presentation of an ambiguous cube. The other blocks were randomly allocated a different type of cue, defined by the 2 × 2 factorial combination of two possible orientations (contradicting or supporting) and two possible strengths (weak or strong).

Participants were separated into four groups (n = 12, 14, 14, and 15) that differed in terms of how we altered their prior knowledge. The first group was presented with a tilted cube, which was expected to neutralize the SFA implicit bias (Figure 1A (4)). The remaining three groups viewed a normal cube—where the implicit prior is deemed present—but received different types of instructions, which we used to manipulate their implicit prior. In group 2, the instructions explicitly mentioned the presence of the implicit bias:

“*When looking at the cube, most people tend to see it with its front side on the right. In other words, a natural tendency exists to see the cube mostly ‘from above’; In the present experiment, we aim to study the characteristics of this spontaneous preference*.”

Because the statement was correct, the instructions were considered to support the spontaneous bias (supporting instructions). In group 3, participants were informed about a natural tendency to primarily perceive the cube as though it were viewed from below. The wording was similar, but the statement was incorrect, thus contradicting the implicit prior (contradictory instructions). In group 4, the participants received no complementary information. In this case, their prior knowledge was considered similar to the implicit bias (neutral instructions). Notably, the difference among the four groups was only the verbal instructions, while all groups received the same visual instructions, including the stimulus and the two possible interpretations, to avoid any additional priming effects. As shown inTable 1, the four groups did not significantly differ in terms of demographic characteristics.

**Table 1.** Demographic characteristics of the 4 groups (without outliers).

					Comparison	
Variables	Tilted (n = 12)	Instr. Supp. (n = 14)	Instr. Contr. (n = 14)	No Instr. (n = 15)	Test	*p*
**Age**	23.33	28.64	28.93	29.27	1.31^*^	0.28
Mean (*SD*)	(2.77)	(7.19)	(9.60)	(11.73)		
**Education**	17.25	19.07	18.57	18.00	1.77^*^	0.16
Mean (*SD*)	(2.42)	(1.94)	(2.17)	(1.96)		
**Sex ratio** (male:female)	3:9	7:7	8:6	9:6	3.87^†^	0.28

The four groups did not differ in terms of age, education, or sex.

* F-test.

† Chi-square test.

Participants were additionally instructed to gaze at a fixation point in the middle of the screen to neutralize the potential confounding effects of eye movements. A training session allowed each participant to familiarize himself/herself with the stimuli and the apparatus.

The experiments were implemented in MATLAB v. 2011b (MathWorks, Natick, MA) using Psychtoolbox v. 3.0.10. Stimuli were displayed on a 17-inch LED screen with a resolution of 1280 × 1024 pixels. Responses were collected using a classical computer keyboard. A chin-cup and forehead bar ensured the immobilization of the participant's head at a distance of 60 cm between the eye and the screen.

### Model-free analysis

#### Measured variable

RP was calculated by determining the grand mean of responses across trials, runs, and participants. It was interpreted as the general probability to perceive one interpretation or the other in each trial. A value of 1 or 0 corresponded to the complete dominance of the SFA or SFB interpretation during perception, respectively. A value of 0.5 would characterize a purely chance level, where the two percepts are equiprobable.

#### Statistical analysis

Because RP is a ranged variable, we exclusively performed non-parametric analyses. The effects of priors, sensory evidence, and their interaction were tested using a linear mixed-effects model comprising the effects of cues and instructions, as well as their interaction as fixed effects, together with Gaussian random effects for intercepts and slopes. For an analysis of significant omnibus effects, we performed post hoc comparisons using either paired or unpaired rank-sum tests to clarify simple effects on the 2 × 2 design. Finally, one-sample Wilcoxon signed-rank tests were performed to compare the mean RPs with values of 0.5 (i.e., the chance level). All significance tests were performed on the final sample of the 55 participants (n = 12, 14, 14, and 15 participants in each group, respectively), the analyses were two-tailed with an alpha value of 0.05 and were performed using the statistical toolbox of MATLAB v. 2011b (MathWorks).

### Model-based analysis

#### Models

We conceptualized perception as an inferential process in which the brain generates a subjective belief about the possible interpretations of the NC (i.e., a posterior probability) and uses it to make a perceptual decision, particularly whether it is an SFA or SFB cube. Three different models were fitted to the average RPs of the four groups. All the models assumed independence between the sequential perceptual decisions within a run. They differed in how the three main effects of the experiment (sensory evidence *S*, an implicit prior *P_impl_*, and an explicit prior *P_expl_*) were combined to generate the posterior probability *P*(*X*|*S*, *P_impl_*,*P_expl_*). In this expression, *X* is a binary variable that corresponds to the 3D interpretation (*X*  =  1 corresponds to SFA, *X*  =  0 corresponds to SFB).

**Figure 3.**
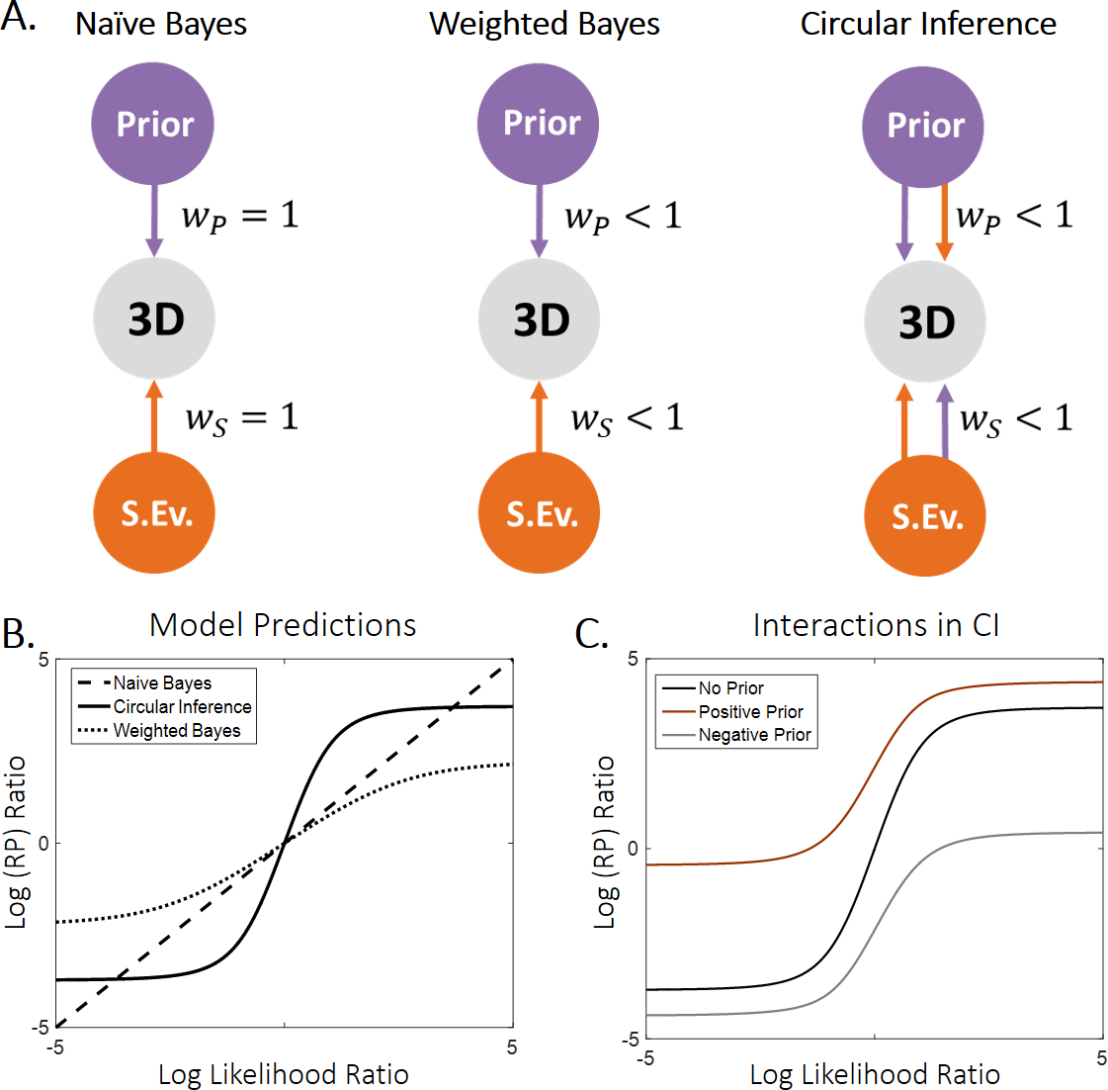
Models and model predictions. (A) Three different models were used to fit the data. The simplest model (naïve Bayes [NB], left panel) consisted of a simple addition of the sensory evidence and prior on the log scale and is equivalent to a three-layer generative model in which all the connections are equal to 1. The weighted Bayes (WB) model (middle panel) further assumes that only partial trust exists between the nodes of the generative model. Importantly, both the NB and WB models do exact inference. Finally, we used a circular inference (CI) model (right panel) that further allows reverberation and overcounting of sensory evidence and prior knowledge. (B) The log(RP) ratio predicted by the models as a function of the log-likelihood ratio. The NB model predicts a linear dependence, whereas both the WB and CI models predict sigmoid curves (due to the saturation imposed by the weights). Furthermore, the three models generate different predictions about the slope of the curves around zero. The NB and WB models predict a slope of 1 and less than 1, respectively, and only the CI model predicts a slope greater than 1. (C) In the CI model, the slope of the log-likelihood/log-posterior curve also depends on the log-prior as a result of the reverberations, indicating an interaction between the two different types of information (Leptourgos et al., 2017). Weaker priors are associated with steeper sigmoid curves. The reason is the saturating effect of the weight when priors and sensory inputs are congruent (they are both positive/negative).

Three different models were used, each implementing a different method of doing hierarchical probabilistic inference. All are based on a message-passing algorithm called Belief Propagation (Bishop, 2006) (see the Supplementary Material – Computational Modeling section for more information about the models). The simplest model that was fitted to the data is the naïve Bayes (NB) model, which assumes perfect integration of likelihoods and priors according to the Bayes theorem. Consequently, it is equivalent to a basic multiplicative rule (Rubén Moreno-Bote, Knill, & Pouget, 2011; Rubén Moreno-Bote, Shpiro, Rinzel, & Rubin, 2008) [additive rule in the log scale] (eq. 1; Figure 3A, left panel). The weighted Bayes (WB) model extended the NB model by assuming only partial trust of the sensory evidence and prior information (eq. 2; Figure 3A, middle panel). Crucially, both models are Bayesian models that generate an exact inference. Finally, the third model is a CI model (Jardri et al., 2017) and the information is not only weighted, as in the WB model, but it is also amplified, because of information loops (eq. 3; Figure 3A, right panel). As a result, the CI model is generating a sub-optimal inference, which renders it qualitatively different from the other two models.

The three models are quantitatively described by the following equations:

(1) $$L_{RP}=L_{S}+L_{impl}+L_{expl}$$

(2) $$L_{RP}=F\left(L_{S},w_{S}\right)+F\left(L_{impl}+L_{expl},w_{P}\right)$$

(3) $$\begin{array}{ccc} \;L_{RP}\; & = & F\left(L_{S}+F\left(L_{S},w_{S}\right)+F\left(L_{Pr},w_{P}\right),w_{S}\right)\\ \; & & \;+F\left(L_{Pr}+F\left(L_{S},w_{S}\right)+F\left(L_{Pr},w_{P}\right),w_{P}\right)\; \end{array}$$

where *F*(*L*, *w*) is a sigmoid function:

(4) $$F\left(L,w\right)=log\left(\frac{we^{L}+1-w}{\left(1-w\right)e^{L}+w}\right)$$

and *L_Pr_* =  *L_impl_*  +  *L_expl_*. *L_RP_* corresponds to the log-ratio of the RP and is equal to the log-posterior ratio. That assumption is based on the hypothesis that perceptual decisions are made using probability matching, a commonly observed strategy in sequential “two-alternative forced-choice” tasks (Daw, O'Doherty, Dayan, Seymour, & Dolan, 2006; Drugowitsch et al., 2016; Rubén Moreno-Bote et al., 2011). The application of Softmax to the log posterior odds (a more appropriate model for perceptual decisions) would only induce a global change in the gain of the former (more information is provided in the Supplementary Material – Softmax Decision Criterion and Supplementary Figure S2) and would not affect any of our conclusions (models equipped with a Softmax decision criterion were also fitted to the data; see Supplementary Figure S3).

(5) $$L_{RP}=log\left(\frac{RP}{1-RP}\right)$$

The log-likelihood ratio *L_s_*, the implicit log-prior ratio *L_impl_* and the explicit log-prior ratio *L_expl_* are calculated using the following equations:

(6) $$L_{s}=log\left(\frac{S}{1-S}\right)$$

(7) $$L_{impl}=log\left(\frac{P_{impl}}{1-P_{impl}}\right)$$

(8) $$L_{expl}=log\left(\frac{P_{expl}}{1-P_{expl}}\right)$$

Because none of these variables were known, they were all treated as free parameters (*L_impl_* is equal to 0 in the case of the tilted cube [group 1] and *L_expl_* is equal to 0 when no explicit instructions are provided [groups 1 and 4]). We further considered symmetry both for the effects of the cues and the instructions to reduce the total number of free parameters needing to be optimized to the greatest extent possible, resulting in four free parameters (*L*_*s*,*strong*_, *L*_*s*,*weak*_, *L_impl_*,*and* *L_expl_*). As a control, we also considered the case of asymmetrical instructions (*L*_*s*,*strong*_, *L*_*s*,*weak*_, *L_impl_*,*L*_*expl*,*SFA*_, *and* *L*_*expl*,*SFB*_) (see Supplementary Figure S3).

Finally, *w_S_* and *w_P_* (appearing only in the WB and CI models) correspond to participants’ trust (or weight) of the sensory evidence and priors, respectively, and constituted the two additional free parameters of those models:

(9) $$\;w_{S}=P\left(X=1|S=1\right)=P\left(X=0|S=0\right)$$

(10) $$\;w_{P}=P\left(X=1|P=1\right)=P\left(X=0|P=0\right)$$

Importantly, because the SFA prior was completely uninformative in the case of the titled cube (the “point of view” (SFA or SFB) does not predict any feature of the configuration of the tilted cube, which remains true even if we are highly confident about our point of view), we considered the following:

(11) $$\;w_{P}>0.5\;if\;normal\;cube,w_{P}=0.5\;if\;tilted\;cube$$

meaning that there is stronger CI in the normal cube condition, in which our beliefs about our point of view affect our beliefs about the configuration of the cube and vice versa (creating an inference loop). As a control, we also considered the case in which *w_P_* has the same value in all conditions (see Supplementary Figure S3).

An illustration of the different models is presented in Figure 3A. The CI model (Figure 3A, right panel) hypothesizes that the perceptual system generates an approximate inference because of the inefficient control of the information that is propagated in the hierarchical network (Jardri & Denève, 2013). That lack of efficient control leads to a failure to efficiently remove redundant messages (i.e., a reverberating prior, which is misinterpreted as sensory evidence, re-ascends the hierarchy and corrupts the likelihood term and redundant sensory evidence, which descends the hierarchy and corrupts the prior term). Additionally, as described in a previous study (Jardri et al., 2017), a cross-term is added to each component, rendering likelihood and prior information completely inseparable. Because of those extra terms, the sensory evidence and prior components become aberrantly correlated and subsequently generate an interaction (Leptourgos et al., 2017). The WB model (Figure 3A, middle panel) was derived from the CI model by removing the reverberated terms, whereas the NB model (Figure 3A, left panel) was generated by further assuming that *w_S_* = *w_P_* =  1.

The CI model used here was similar to the model used by Jardri and colleagues to explain participants’ behaviors (both individuals suffering from schizophrenia and healthy participants) in a probabilistic reasoning task (Jardri et al., 2017). Nevertheless, an important difference must be highlighted. In the present study, the redundant messages corrupted the original messages only once (overcounting of information still occurred, but the amount of amplification was constrained), which is equivalent to setting *a_S_* and *a_P_* (the parameters in the original model that represented the number of times the redundant messages were considered) equal to 1. We had two reasons for constraining the values of these parameters. First, fixing the number of loops did not qualitatively change the results. Indeed, the resulting model predicted both a slope greater than 1 and an interaction between sensory evidence and priors, the two characteristic features of circular inference observed in the data. Second, the additional complexity (two additional free parameters) did not further improve the fit (see Supplementary Figure S3).

Figure 3B illustrates the predictions of the three models. In contrast to the linear NB model, both the WB model and the CI model are non-linear models, due to the saturation of the posterior that is caused by the weights. Importantly, the three models generate different predictions about the slope of the log-likelihood/log-posterior curve around 0: the NB model and WB model predict a slope equal to and less than one, respectively. Interestingly, only the CI model generates a slope that is greater than one, due to its overcounting of the prior and of sensory evidence (e.g., if we assume *L_impl_* = *L_expl_* =  0 and *w_S_* = *w_P_* =  1,  eq. 3 becomes *L_RP_* =  3*L_S_*, indicating that the sensory input is counted three times instead of one). Moreover, it predicts an interaction between the prior and sensory evidence such that the slope differs, depending on the prior strength and weight (Figure 3C).

Finally, in eqs. 1-3, we assumed that the instructions act as an additional prior term, essentially altering the strength of the implicit preference independently of the presence of a visual cue. As a result, any interaction between the effect of the cue and the effect of the instructions is forbidden under Bayesian formalisms and is only explained by non-Bayesian mechanisms, such as the presence of circular inference. Notably, alternative interpretations of the instructions (which are even more complex) might also generate this interaction, particularly likelihood-dependent instructions or instructions that directly affect the reliability of the sensory evidence. Those additional models were also considered and compared to the CI model (see Supplementary Figure S3).

#### Model fitting

All the models were fitted to the data by minimizing the mean squared distance between the log(RP) ratio for the different conditions and the predictions of the models. Instead of simply considering the means, we used data points from each participant, completely using the available information but assuming that the parameters did not vary between participants. The optimal values for parameters were obtained using a non-linear programming method (sequential quadratic programming; a built-in MATLAB function) that is appropriate for non-linear constrained multivariable functions. The optimization process was repeated 100 times for each model, with initial values chosen each time randomly from the parameter space, to avoid local minima. The robustness of the results was evaluated using a “Jackknife” resampling method (Efron & Stein, 1981), which consists of refitting our models to all the possible subsamples of size (N-1) (sequentially deleting one participant from our initial sample of size N = 55; the total number of subsamples is equal to the initial sample size N) and recalculating the Bayesian information criterion (BIC) scores.

#### Model comparison

We compared the quality of the fits for the three models using BIC scores. We approximated the likelihoods of all the models as normally distributed. The BIC score was then calculated using the following equation:

(12) $$BIC=nlog\left(\sigma^{2}\right)+klog\left(n\right)$$

where *n* is the total number of data points (5 points per participant), σ^2^ is the mean squared error, and *k* is the number of free parameters (4 for the NB model and 6 for the other models).

## Results

### Model-free analysis

**Figure 4.**
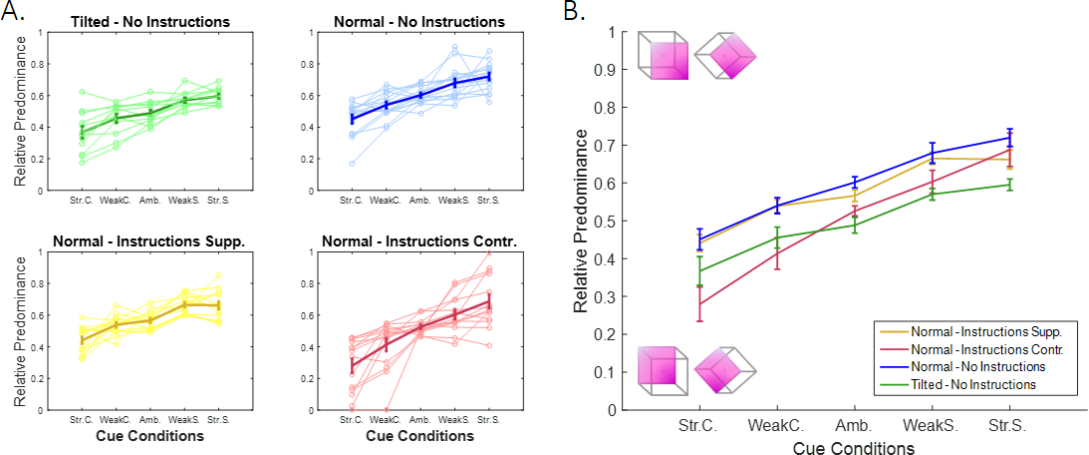
Relative predominance between conditions. (A) The four subplots illustrate the four different prior conditions: tilted cube (top left plot, green; n = 12) or normal cube with no instructions (top right plot, blue; n = 15), supporting instructions (bottom left plot, yellow; n = 14) or contradictory instructions (bottom right plot, red; n = 14). The x-axis presents the five cue conditions, ranging from a strong cue supporting the SFB interpretation (left panels) to a strong cue supporting the SFA interpretation (right panels). Thin lines correspond to the behaviors of single participants (outliers are not presented), and thick lines represent the average RP for each group calculated after removing the outliers (±  SE). (B) Between-groups comparison of average RP values. A linear mixed-effects model revealed significant effects of sensory evidence (*p* < 0.001) and the prior (contradictory instructions, *p* < 0.001) and tilt (*p* < 0.001) manipulations. We also observed a cue x instruction interaction for the contradictory instructions (red curve) compared with supporting instructions (yellow curve, *p* = 0.016) and the tilted cube (green curve, *p* = 0.021).

The effects of prior knowledge and the manipulations of sensory evidence are presented in Figure 4. RP values were not significantly different between the 2 ambiguous blocks (runs 1-5 and 16-20) in any of the groups (*p* > 0.1), indicating only minor effects of fatigue (at least until the 20th run) and a stable effect of the instructions. The manipulation of sensory evidence significantly affected bistability, with RP increasing as the visual cue changed from strongly contradicting to strongly supporting (*β* = 0.415, *p* < 0.001). The manipulation of prior knowledge through the instructions only affected RP in the case of contradicting instructions, with a significant overall reduction in RP (*β* = -0.096, *p* < 0.001). Tilting the cube in the absence of any instruction resulted in a significant decrease in RP (*β* = 0.103, *p* < 0.001), which substantiated the effect of an implicit prior that naturally biases perception toward SFA dominance (the RP in the case of a tilted cube – ambiguous condition was not significantly different from chance, *p* > 0.05). Importantly, we identified a significant interaction between the continuous effect of cue and the effect of contradicting instructions (compared to the normal cube with supporting instructions and the tilted cube with no instructions; *β* = 0.265, *p* = 0.016 and *β* = 0.265, *p* = 0.021, respectively). This interaction should not be present for a purely Bayesian observer because the contribution of sensory evidence and priors (when expressed as the log odds ratio) should be additive.

### Model-based analysis

**Figure 5.**
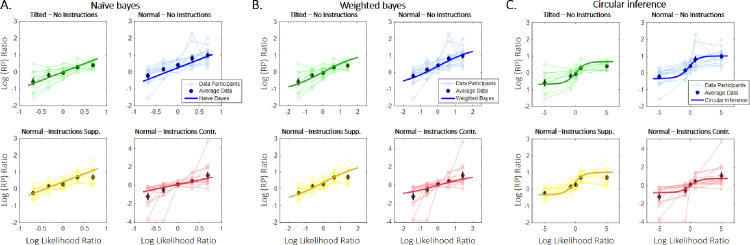
Observed and predicted log(RP) ratios as a function of the log-likelihood ratio. Different colors correspond to different prior conditions. Thin lines represent data from single participants, highlighted points correspond to average RPs (± SE), and thick lines illustrate the predictions generated by the models. The three models are presented separately, since the likelihood was itself considered a free parameter [(A): NB, (B): WB, and (C): CI]. The models were fitted to aggregated data from all participants by minimizing the mean squared distance between the observed and predicted log(RP) ratios.

Figure 5 illustrates the best-fitting NB (5A), WB (5B), and CI models (5C) and the values of the free parameters in the three models are presented in Supplementary Figure S1. The three models predicted very different values for likelihoods and priors. These differences were easily explained by the assumption of perfect trust in sensory evidence and priors in the NB model, whereas the other two models predict much lower weights (*w_S_* =  0.77, *w_P_* =  0.59 for the WB model and *w_S_* =  0.66, *w_P_* =  0.59 for the CI model).

The NB model qualitatively captures most trends in the data (see also Supplementary Figure S5), with the following exceptions. First, it underestimates RP in the case of the normal cube without instructions (Figure 5A, blue curve), and second, it is unable to predict the correct slopes. The latter limitation is particularly striking in the case of a normal cube with contradicting instructions, where the slope is larger than predicted (i.e., >1; Figure 5A, red curve). The WB model performs better than the NB model under most conditions, but it also underestimates the effect of the cue when the instruction contradicts the SFA preference (see Figure 5B, red curve). In contrast, the CI model captures this last trend (see Figure 5C), suggesting that the variability of the cue effect (the slope) under different conditions is due to circularity in the inference process. CI also explains the asymmetry between supporting and contradictory instructions (the latter but not the former exerts a significant effect on RP), without adding new free parameters (e.g., asymmetrical explicit prior *L_expl_*). Supporting instructions push the prior belief toward stronger positive values. It thus falls into the saturating part of the sigmoidal curve (induced by the non-linear “factors” F). In this range, an increase in the prior strength exerts little effect on the posterior. In contrast, contradictory instructions bring the total prior closer to zero, where the slopes of the sigmoid are larger. This shift results in a stronger effect of the contradicting instructions, without requiring any asymmetry in *L_expl_* (Supplementary Figure S4).

**Figure 6.**
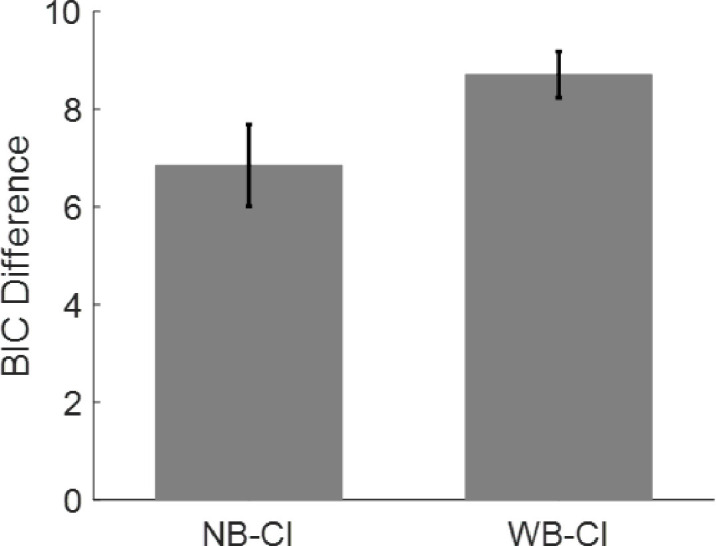
Comparison of the three models. The CI model outperforms both the NB and WB models (note that a positive difference indicates lower BIC score for CI and thus better performance). Fitting was repeated multiple times, and one of the participants was removed each time (“Jackknife” resampling method). In all cases, (55 possible subsamples), the CI model outperformed the other two models by producing a difference in BIC scores greater than 4.5, whereas in 48/55 cases, the difference was greater than 6. Error bars correspond to standard deviations of the jackknife estimates.

A quantitative comparison of the three models using BIC scores, which penalizes the use of extra free parameters in the WB and CI models, indicated that the CI model significantly outperformed the two Bayesian models (Figure 6). A lower BIC score indicates that the model provides a better fit for the data, with a difference >2 considered positive and a difference >6 considered strong (Kass & Raftery, 1995). A “Jackknife” resampling method was used to evaluate the robustness of those results. In all cases (N = 55 possible subsamples), the CI model outperformed the other two models by presenting a difference in BIC scores >4.5, whereas in 48/55 cases, the difference was >6.

## Discussion

The goal of the current study was to decipher how priors and sensory evidence are combined to shape bistable perception. We particularly wished to investigate whether this integration is probabilistically optimal or if other principles are involved, contributing to the debate on whether bistable perception is a by-product of perceptual inference (regardless of its neural implementation). Our results suggest an imperfect neural implementation of probabilistic inference, possibly due to an imbalance between excitation and inhibition in neural circuits.

Consistent with a previous report (Dobbins & Grossmann, 2010), we observed asymmetry in the way participants interpreted the completely ambiguous NC. This finding supports an implicit preference (implicit prior) to perceive objects in an SFA configuration (Mamassian & Landy, 1998). More surprisingly, this preference was explicitly manipulated by providing the participants information that either confirmed or rejected it (explicit prior). Consistent with previous studies (Klink, van Ee, & van Wezel, 2008; Levelt, 1967; Mamassian & Goutcher, 2005), the addition of visual cues also significantly biased perception toward the corresponding interpretation. The qualitative effects of implicit priors, explicit priors and sensory evidence appeared compatible with a probabilistic combination of information, suggesting that a Bayesian inference was still involved.

However, we also observed a significant interaction between priors and sensory evidence that was not explained by the exact inference. In particular, the effect of sensory cues was stronger when the prior was more ambiguous (e.g., when the implicit preference for SFA was contradicted by instructions) and weaker in the absence of a prior (e.g., a tilted cube). In contrast, Bayes’ theorem predicts that sensory cues are weighted according to their reliability, independently of the prior. After comparing the parametric models, the present data were better represented by a CI model, in which prior beliefs (i.e., the instructions and SFA preferences) corrupt new sensory evidence (i.e., ambiguous cues are misinterpreted as supporting the current belief) and vice versa (CI is the simplest model that is able to explain all the main features of the data; more complex models (e.g., assuming different softmax temperature β per group and asymmetrical instructions) might have a similar explanatory power but contain far too many free parameters). This corruption could be the result of feedback signals to sensory areas, that are insufficiently controlled by inhibition (Jardri & Denève, 2013). This feedback might also cause multistable perception (i.e., generate a bistable attractor; see Supplementary Figure S7) by temporarily stabilizing the current percept, despite the absence of supporting evidence (Leptourgos et al., 2017).

These findings add new elements to a long-lasting debate in neuroscience that questions whether perception is mainly driven by bottom-up processes or whether top-down effects are equally important (Heeger, 2017). Multiple studies have investigated how low- or high-level manipulations affect bistability, without offering definitive answers. For the former, authors have used priming or suppressing effects (usually attributed to adaptation) (Kanai & Verstraten, 2005; Nawrot & Blake, 1989; Joel Pearson & Brascamp, 2008; Joel Pearson & Clifford, 2005), changes in retinal location (Long & Toppino, 2004), manipulation of the type of presentation (continuous–intermittent) (Leopold, Wilke, Maier, & Logothetis, 2002; Orbach, Ehrlich, & Heath, 1963), and direct manipulation of the properties of the stimulus, such as the intensity (Lynn, 1961) and completeness (Babich & Standing, 1981). In contrast, studies of high-level manipulations have focused on the effects of volition (Toppino, 2003; Van Ee, Van Dam, & Brouwer, 2005), expectation and prediction (Denison, Piazza, & Silver, 2011), attention (Chong & Blake, 2006; Dieter & Tadin, 2011; Stonkute, Braun, & Pastukhov, 2012), learning (Haijiang, Saunders, Stone, & Backus, 2006), mental imagery (Pearson, Clifford, & Tong, 2008), knowledge of reversibility (Rock, Hall, & Davis, 1994), and finally the preference for stimuli with a statistical structure similar to natural images (Baker & Graf, 2009; Dobbins & Grossmann, 2010; Zhou, Zhang, Liu, Yang, & Qu, 2010). However, the present study was not designed to test specific neural mechanisms, such as adaptation and noise.

Consistent with the findings from the present study, some authors have focused on how these various effects are combined (Díaz-Santos et al., 2015; Intaite, Noreika, Šoliunas, & Falter, 2013; Kornmeier, Hein, & Bach, 2009). According to Moreno-Bote et al., cue combinations in a bistable display are well explained by a multiplicative law (their predictions are similar to the NB model described here) (Moreno-Bote et al., 2011; the same group proposed that bistability is a form of exploration (Moreno-Bote, Shpiro, Rinzel, & Rubin, 2010)), whereas Zhang and colleagues reported that different types of priors are effectively combined (Zhang et al., 2017). Here, we have extended our study a step further and investigated how top-down (prior manipulation) and bottom-up (sensory cues) effects interact. Rather than inducing a prior through learning, as is widely performed in the literature (Haijiang et al., 2006; Pearson et al., 2008), we directly manipulated participants’ expectations. This manipulation assumes that instructions generate a high-level prior that affects perceptual processing in a manner similar to a learned prior (Schmack et al., 2013).

Despite the amount of available data and the apparent simplicity of the problem, very few published studies have applied normative explanations for bistable perception that include data fitting (Moreno-Bote et al., 2011). Although a proposal of a complete model of bistable perception based on circular inference is beyond the scope of this paper, our current results suggest that a local message passing algorithm with the addition of information loops might constitute the basic principle of such a normative model. Some alternative normative models have relied on a simplified form of Markov Monte-Carlo sampling. More precisely, they assumed that the current percept is based on taking one sample from the posterior distribution and using this sample as a prior for the next time step (Gershman, Vul, & Tenenbaum, 2012; Sundareswara & Schrater, 2008). However, Markov Monte-Carlo sampling requires very long sampling times (because of the temporal correlation between samples) to generate an accurate inference. A possible argument in favor of circular inference would be its ability to quickly and accurately reach correct conclusions in most perceptual tasks, except for particularly ambiguous cases (Jardri & Denève, 2013), making circular inference a powerful model of perceptual inference in unambiguous cases.

From a methodological perspective, and in contrast to most studies on bistable perception in which participants continuously report the dominant percept with a sustained button press (Brascamp, van Ee, Pestman, & van den Berg, 2005; Pastukhov & Braun, 2011), we asked participants to respond discontinuously after being exposed to a go-signal (Mamassian & Goutcher, 2005). This procedure has two main advantages. First, it minimizes the role of attention. Indeed, attention plays a crucial role in bistable perception, particularly the perception of certain bistable stimuli (Li, Rankin, Rinzel, Carrasco, & Heeger, 2017; Toppino, 2003). The inability to control for differences in attentional load between participants potentially represents an important source of uncertainty and even partially explain the substantial variability that has frequently been observed in some publications (see (Mamassian & Goutcher, 2005)). Second, this procedure is less affected by differences in reaction times, as one could use the time of the sound as a proxy for the time of the decision. Consequently, discrete sampling not only appears to be an ideal method for a rigorous experimental exploration of bistable perception but is also useful for adapting this task to specific clinical populations with well-known attentional and motor problems.

We have argued in our previous studies that circularity (and, consequently, the observed interactions between sensory inputs and prior knowledge) potentially result from an imperfect tuning between excitatory and inhibitory signaling in cortical and subcortical circuits (Jardri & Denève, 2013; Jardri et al., 2016; Leptourgos et al., 2017). Indeed, based on extensive evidence, an E/I imbalance in favor of excitation is a central neurophysiological impairment in patients with schizophrenia (Foss-Feig et al., 2017; Jardri et al., 2016), a psychiatric disorder that has been linked to CI (Jardri & Denève, 2013; Jardri et al., 2017). At the implementation level, various mechanisms have been suggested, including dysfunctions of local interneurons in cortical microcircuits (Lewis, Pierri, Volk, Melchitzky, & Woo, 1999) or a dysconnectivity within long-range inhibitory loops (Murray & Anticevic, 2016). In both cases, reverberations (and interactions) occur on a fast timescale, within a few tens/hundreds of milliseconds (timescale of a single trial). Additionally, those processes also accumulate over multiple trials and are driven by the inherent dynamics/persistent activity of the different neuronal populations (this dynamical aspect has been largely neglected in the present paper and will be the focus of future studies). Importantly, unequivocal evidence linking CI to an E/I imbalance is currently lacking, and other implementations might also be involved.

Finally, some limitations must be acknowledged. First, because of the type of priors used (instructions), we were obligated to use a between-subjects design, which prevented us from comparing the effects of different instructions on the same participant. As a result, only five conditions were analyzed per participant, and we were only able to fit our models to averaged data, ignoring variability between participants (see also (Ernst & Banks, 2002; Moreno-Bote et al., 2011). Second, all the models considered here were based on an assumption of temporal independence between the percepts at the time of the sounds. This assumption was partly justified by the weak autocorrelation of the averaged data (see Supplementary Figure S8), although these autocorrelations may be stronger in individual participants (Sundareswara & Schrater, 2008). Nevertheless, temporal statistics would not affect the qualitative predictions of the models (Moreno-Bote et al., 2011). In particular, temporal statistics without circular inference would not provide a valid alternative to the present findings, including the slopes and the cue   × instruction interaction. Third, a response bias might partially account for the effects of the instructions (explicit priors). However, a response bias would exert a similar effect on responses across different cue conditions, while not altering perceptual processing. Although the aforementioned possibility represents one interpretation of the data, it remains highly improbable, given the non-linear interaction observed between instructions and visual cues (see also Supplementary Figure S9 for additional arguments). Finally, although CI was the winning model in all the model comparisons that we implemented (Figures 6 and S3), in certain cases it was only marginally better (e.g. when assuming Softmax with different β parameters across groups). Future studies, possibly involving larger samples, neural data, and testing different predictions of the CI framework (see Figure S7; Leptourgos et al., 2017), are necessary in order to arbitrate between those alternatives and decipher the exact role of circularity in (bistable) perception.

Overall, this study confirms that circular inference is observed in healthy individuals to a certain extent. This unprecedented observation prompts a range of crucial questions that suggest opportunities for further research: in what other ways would circularity affect cognition, and what are its neural substrates? Crucially, we must determine under what circumstances circular inference generates aberrant beliefs or percepts, such as those observed in pathological (neurological or psychiatric) contexts.

## Supplementary Material

Supplement 1

Supplement 2

Supplement 3

Supplement 4

Supplement 5

Supplement 6

Supplement 7

Supplement 8

Supplement 9

Supplement 10
