# Supplementary material for: Circular inference in bistable perception
Source: J Vis. 2020 Apr 21;20(4):12. doi: 10.1167/jov.20.4.12 (PMC7405786; doi:10.1167/jov.20.4.12)
Supplement: Supplement 1 [file jovi-20-4-12_s001.pdf]

Figure S1.

Optimal values of free parameters in the three models [(A): NB, (B): WB, and (C): CI].

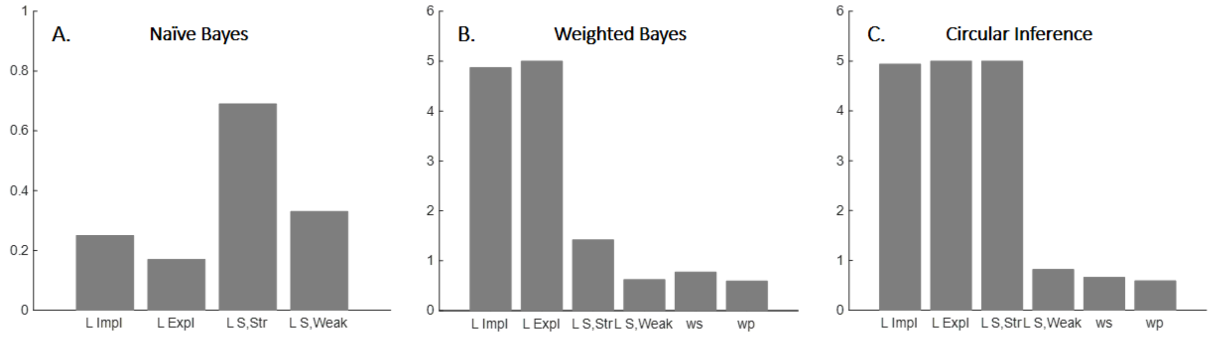

The parameters correspond to the curves presented in **Figure 5** (all the participants). The NB model had fewer free parameters than the other 2 models, since the two weights were by definition set to 1. We observed important differences in the values of the likelihoods ($L_{S,Str}$ and $L_{S,Weak}$) as well as in the values of the priors (L_{impl} and L_{expl}) between the NB model, on one hand, and the WB and CI models, on the other hand. These differences were mainly due to different values of the weights (w_S and w_P). In particular, the CI and WB models provide a more elaborate (but also more accurate) description of the inference problem. We must distinguish the interpretation of the weights, and the interpretations of L_{impl}, L_{expl} and L_S as referring to completely different things. The weights express the trust given by the subject to each type of information, while the likelihoods express the level of reliability of this information.
