# Supplementary material for: Circular inference in bistable perception
Source: J Vis. 2020 Apr 21;20(4):12. doi: 10.1167/jov.20.4.12 (PMC7405786; doi:10.1167/jov.20.4.12)
Supplement: Supplement 2 [file jovi-20-4-12_s002.pdf]

Figure S2.

Model predictions in the case of a Softmax Decision Criterion.

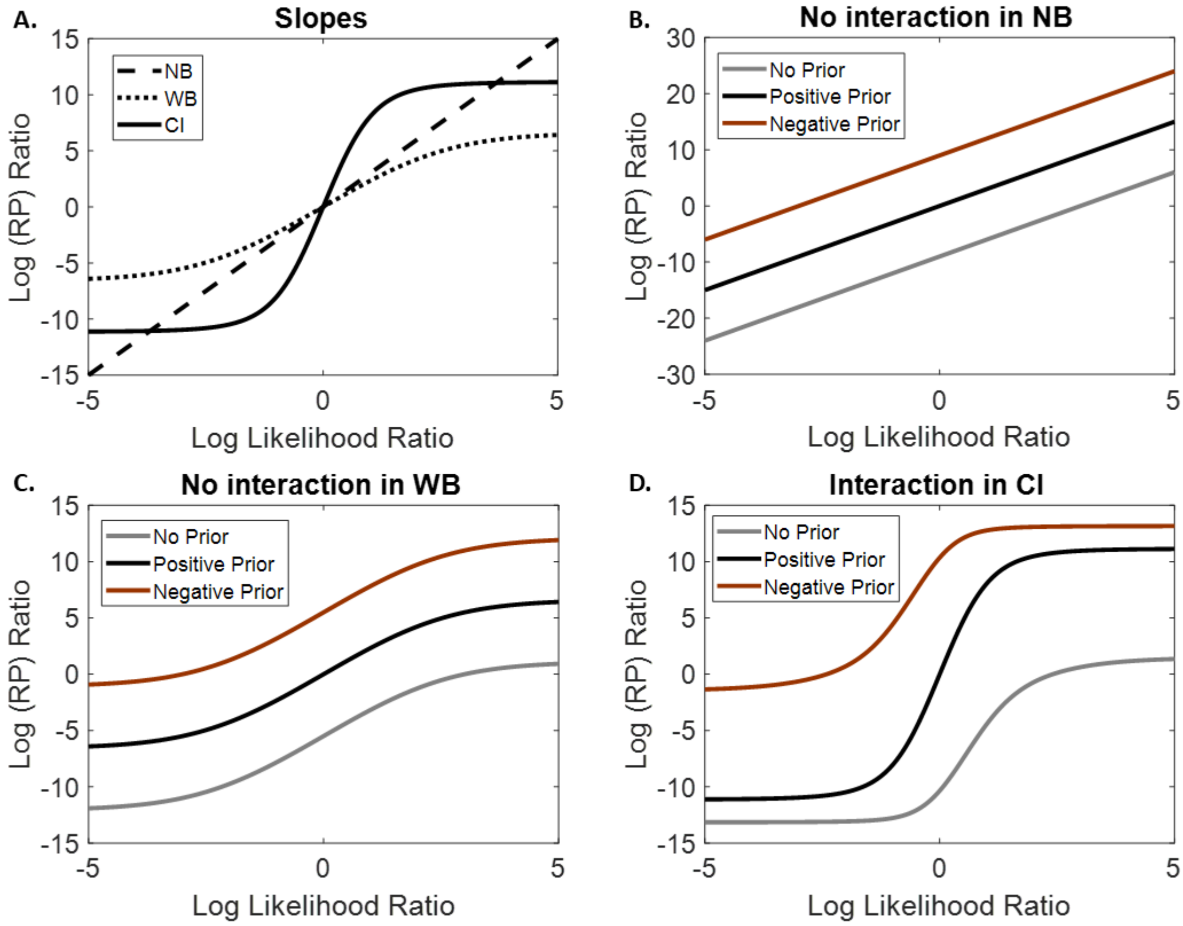

In the **Main Text,** we presented models in which decisions are made through probability matching (the probability of choosing SFA interpretation is equal to the posterior probability). Another way of making decisions is through Softmax (eq. $(S10), (S11)$). Probability matching, is a special case of Softmax (for $\beta = 1$). Softmax only adds a multiplicative term in the equations (eq. $(S13) - (S15)$). Although the additional parameter β is able to generate slopes larger than 1 (**panel A.**), it cannot account for the difference in slopes between the different groups without the addition of circularity (**panels B-D**).
