# Supplementary material for: Circular inference in bistable perception
Source: J Vis. 2020 Apr 21;20(4):12. doi: 10.1167/jov.20.4.12 (PMC7405786; doi:10.1167/jov.20.4.12)
Supplement: Supplement 3 [file jovi-20-4-12_s003.pdf]

Figure S3.

Additional model comparisons.

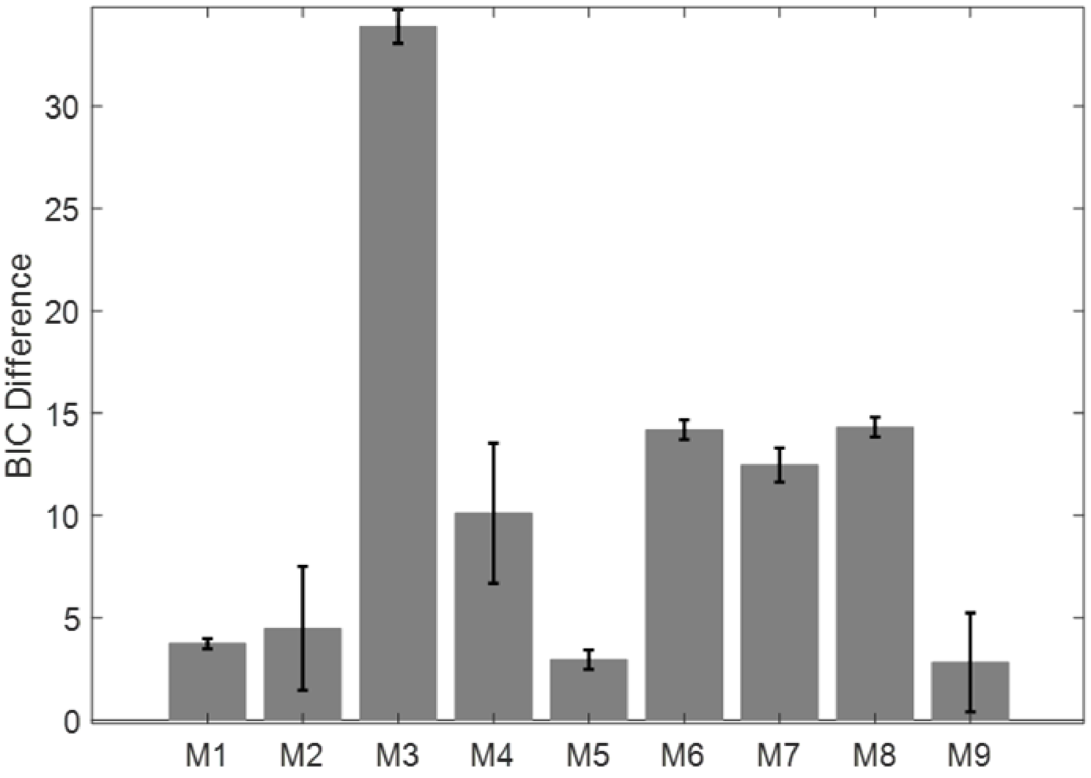

All the models were compared against the CI model presented in the **Main Text**. Positive values denote a CI win. Differences above 2 are considered as positive; differences above 6 are considered as strong. Error-bars (Jackknife resampling method; see **Main Text**) correspond to standard deviations of the Jackknife estimates. **M1: Model assuming different effects of priors in the case of the tilted cube**. In the **main text**, we assumed that $w_P = 0.5$ when the cube is tilted, since the prior becomes uninformative and thus irrelevant in this case. An alternative would be that the prior weight remains greater than 0.5 (as in the case of the normal cube), and that only the log-prior ratio becomes equal to zero. The 2 alternatives are unable to be differentiated by the NB and WB models, but they result in different predictions when the CI model is used, in which a reverberating likelihood term appears inside the prior term. This reverberating term disappears completely if we assume that $w_P = 0.5$, whereas it remains if we assume that $L_P = 0$. **M2: Circular inference models with fixed and optimized loops**. In a previous study, Jardri and colleagues considered a *CI model* in which the strength of climbing loops (reverberation of sensory evidence, a_S) and the strength of descending loops (reverberation of priors,

a_P) were considered free parameters and were optimized (Jardri et al., 2017). The predictions of the model were summarized by the following equation: $L_C = F(L_S + F(a_c L_S, w_S) + F(a_d L_P, w_P), w_S) + F(L_P + F(a_c L_S, w_S) + F(a_d L_P, w_P), w_P)$. In the current study, we fixed the values of these 2 extra parameters to 1, obtaining equation (1) (**Main Text**). The two models make the same qualitative predictions, as they both contain reverberating terms that render likelihood and prior inseparable. **M3: WB model in which the instructions are likelihood-dependent.** A likelihood-dependent effect of the instructions may constitute an alternative explanation for the interaction observed between the effect of the visual cues and that of the instructions/priors. In our framework, such an interpretation can be implemented as follows: $L_{RP} = F(L_S, w_S) + F\left(L_{impl}, w_{P,impl}\right) + F(L_{expl}, w_{P,expl}(L_S))$, where the weight attributed to the instruction is likelihood-dependent. Despite its plausibility, such an implementation drastically increases the complexity of the model, since it comprises 9 free parameters (instead of w_P , we now have [$w_{P,impl}$, $w_{P,expl}\left(L_{S,amb}\right), w_{P,expl}\left(L_{S,Str}\right), w_{P,expl}(L_{S,weak})$]). **M4: WB model in which the instructions directly affect the reliability of the visual cue.** The observed interaction between visual cues and priors could be explained by assuming that the instructions do not act as a prior but instead change the reliability of the sensory evidence. In our framework, such an interpretation could be implemented as follows: $L_{RP} = F(L_S, w_S(Instr)) + F\left(L_{impl}, w_P\right)$, where the sensory weight depends on the instructions. This model comprises 7 free parameters. **M5 - M6: Models (NB, WB) with asymmetrical instructions.** In the **Main Text** we made the assumption that instructions are symmetrical ($L_{expl,SFA} = -L_{expl,SFB}$). As a control, we also fitted models with asymmetrical instructions (different free parameters for SFA and SFB instructions). **M7 – M8: Models (NB, WB) with a Softmax decision criterion** (parameter β was fixed across groups). **M9: WB with Softmax, with different $\boldsymbol{\beta}$ parameters across groups (4 additional free parameters).** It is worth noting that if instructions (and tilting) had an effect on β, the interaction could exist even without circularity. A NB model (with Softmax; different β)outperformed a CI model using Probability Matching (our baseline model in this study), but not more complex CI models (with Softmax etc. – not shown here).
