# Supplementary material for: Circular inference in bistable perception
Source: J Vis. 2020 Apr 21;20(4):12. doi: 10.1167/jov.20.4.12 (PMC7405786; doi:10.1167/jov.20.4.12)
Supplement: Supplement 4 [file jovi-20-4-12_s004.pdf]

Figure S4.

CI can account for the asymmetrical effect of the instructions.

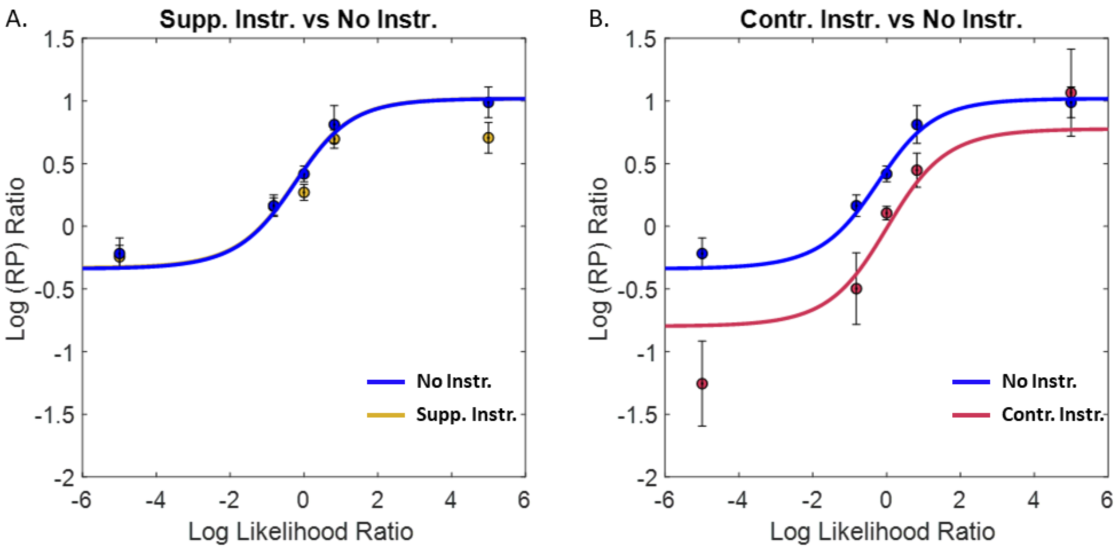

A striking but counterintuitive result is the asymmetrical effect of the instructions. More particularly, SFB (red dots – panel B ± SE) but not SFA instructions (yellow dots – panel A. ± SE) had a significant effect on RP. Interestingly, this is a natural consequence of CI (solid curves). Supporting instructions push the prior belief towards stronger positive values. It thus falls into the saturating part of the sigmoidal curve (induced by the non-linear "factors" F). In this range, an increase in the prior strength has almost no effect on the posterior. On the contrary, contradictory instructions bring the total prior closer to zero, where the slopes of the sigmoid are larger. This results in a stronger effect of the contradicting instructions, without requiring any asymmetry in L_{expl}.
