# Supplementary material for: Circular inference in bistable perception
Source: J Vis. 2020 Apr 21;20(4):12. doi: 10.1167/jov.20.4.12 (PMC7405786; doi:10.1167/jov.20.4.12)
Supplement: Supplement 5 [file jovi-20-4-12_s005.pdf]

Figure S5.

NB captures well the effect of cues.

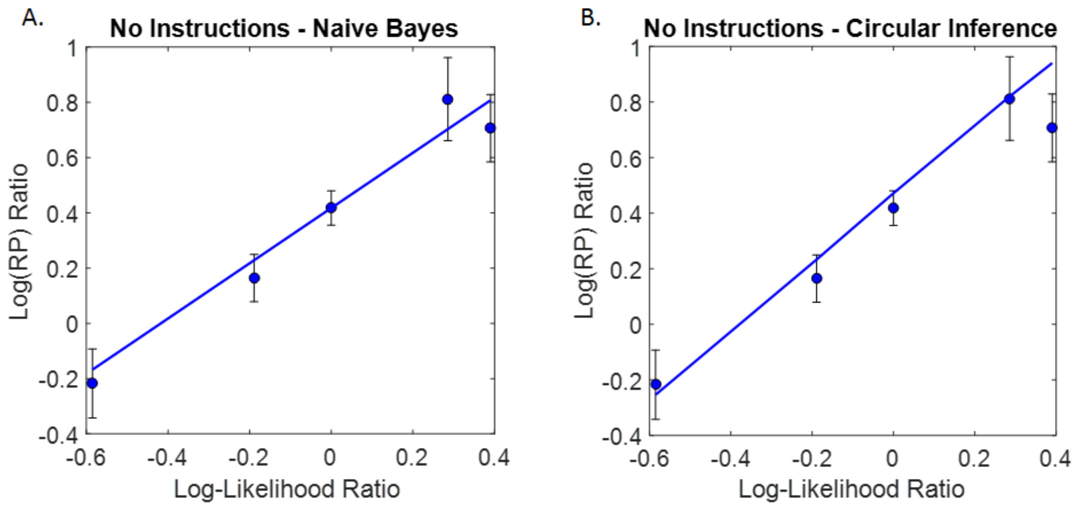

Previous studies showed that cue combination in a bistable perception context is well predicted by a multiplicative rule (Moreno-Bote, Knill, & Pouget, 2011). In agreement with this work, we found that a NB model (i.e. a multiplicative rule) makes a fairly good prediction of the values of the RP in the case of a normal cube with cue (combination of visual cues with implicit priors – **panel A.**; the assessment of the quality of the fit is based on a visual inspection). However, a CI model (**panel B.**) makes a better prediction (based on BIC scores) even when considering only those 4 no-instruction / cue-condition points (BIC scores: NB = -17.44; CI = -21.85 ± SE).
