# Supplementary material for: Circular inference in bistable perception
Source: J Vis. 2020 Apr 21;20(4):12. doi: 10.1167/jov.20.4.12 (PMC7405786; doi:10.1167/jov.20.4.12)
Supplement: Supplement 6 [file jovi-20-4-12_s006.pdf]

Figure S6.

Experimental results after excluding only the 7 outliers who also exhibited qualitatively strange behavior (flat psychometric curves or curves with negative slope or extreme values of RP in the weak-cue or ambiguous condition).

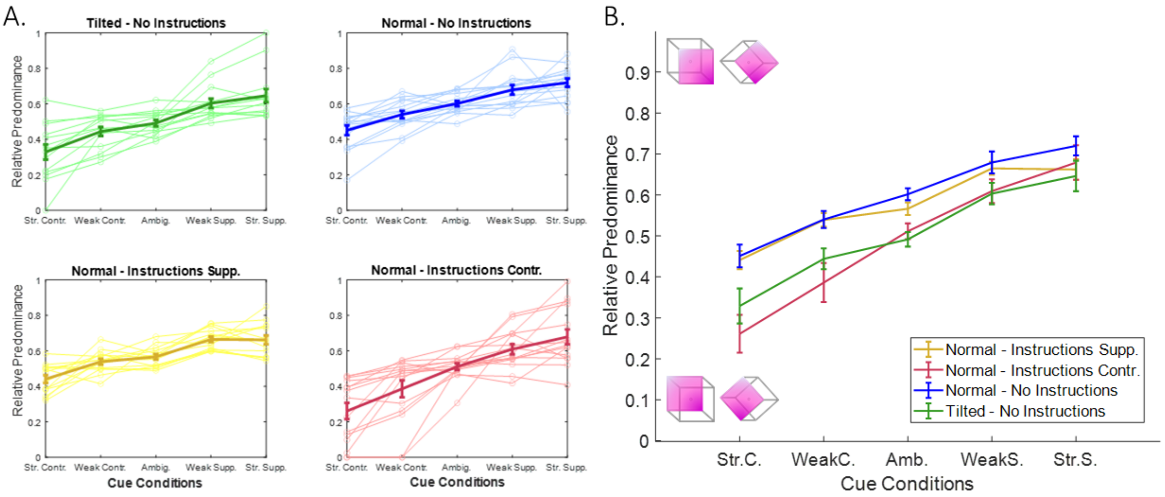

All the results presented in the **Main Text** remained unchanged (Interaction: **p < 0.001**; BIC Scores: NB = -160.88; WB = -159.20; CI = -165.56 ± SE).
