# Supplementary material for: Circular inference in bistable perception
Source: J Vis. 2020 Apr 21;20(4):12. doi: 10.1167/jov.20.4.12 (PMC7405786; doi:10.1167/jov.20.4.12)
Supplement: Supplement 7 [file jovi-20-4-12_s007.pdf]

Figure S7.

Descending loops can generate a bistable attractor.

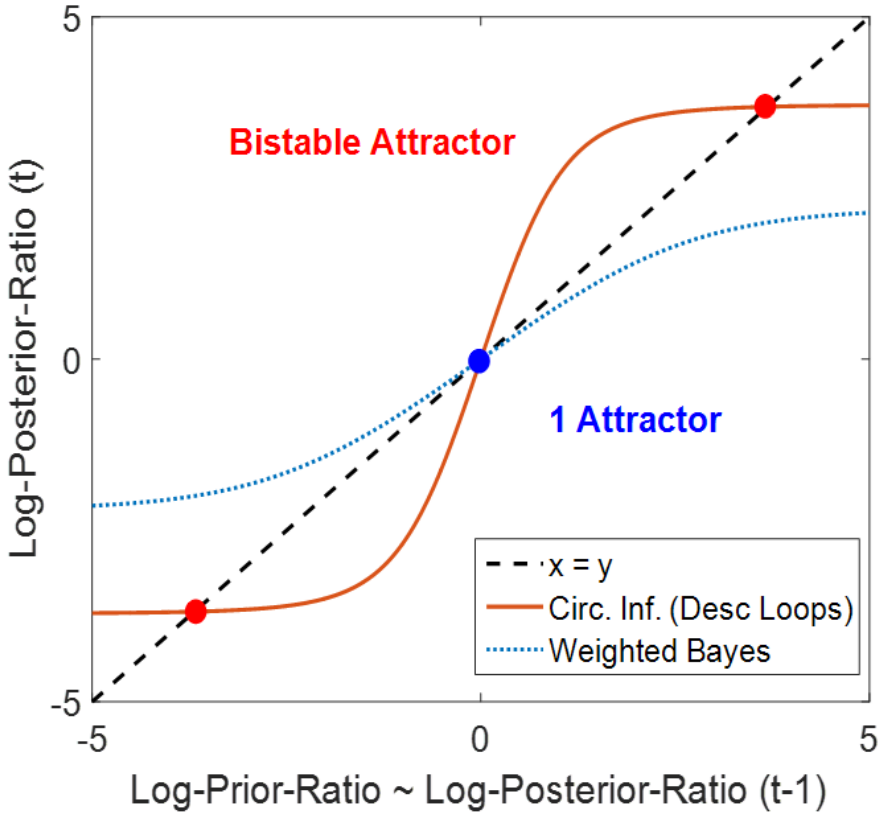

The present results suggest that an aberrant correlation between sensory evidence and priors might be at play in bistable perception, shaping which interpretation we see and when. In the same context, it is important to highlight that a *Circular Inference* model, but not a purely Bayesian model (e.g., the WB model), seems compatible with the phenomenology of bistable perception. When taking into account the dynamics, descending loops (i.e., amplifying accumulated data) introduce a positive feedback to the system, which generates a bistable attractor (2 stable states consisting of strong beliefs, one for each interpretation; red solid line). In contrast, a Bayesian model corresponds to a leaky integrator, in which the belief is similar to chance (blue dotted line) (Leptourgos et al., 2017) and appears unable to generate bistability.
