# Supplementary material for: Circular inference in bistable perception
Source: J Vis. 2020 Apr 21;20(4):12. doi: 10.1167/jov.20.4.12 (PMC7405786; doi:10.1167/jov.20.4.12)
Supplement: Supplement 8 [file jovi-20-4-12_s008.pdf]

**Figure S8.**

**Average autocorrelation functions for the different cue conditions (different subplots) and the different prior conditions** [**(a)**: tilted group (N=12); **(b)**: normal cube, no instructions (N=15); **(c)**: normal cube, supporting instructions (N=14); **(d)**: normal cube, contradictory instructions (N=14)].

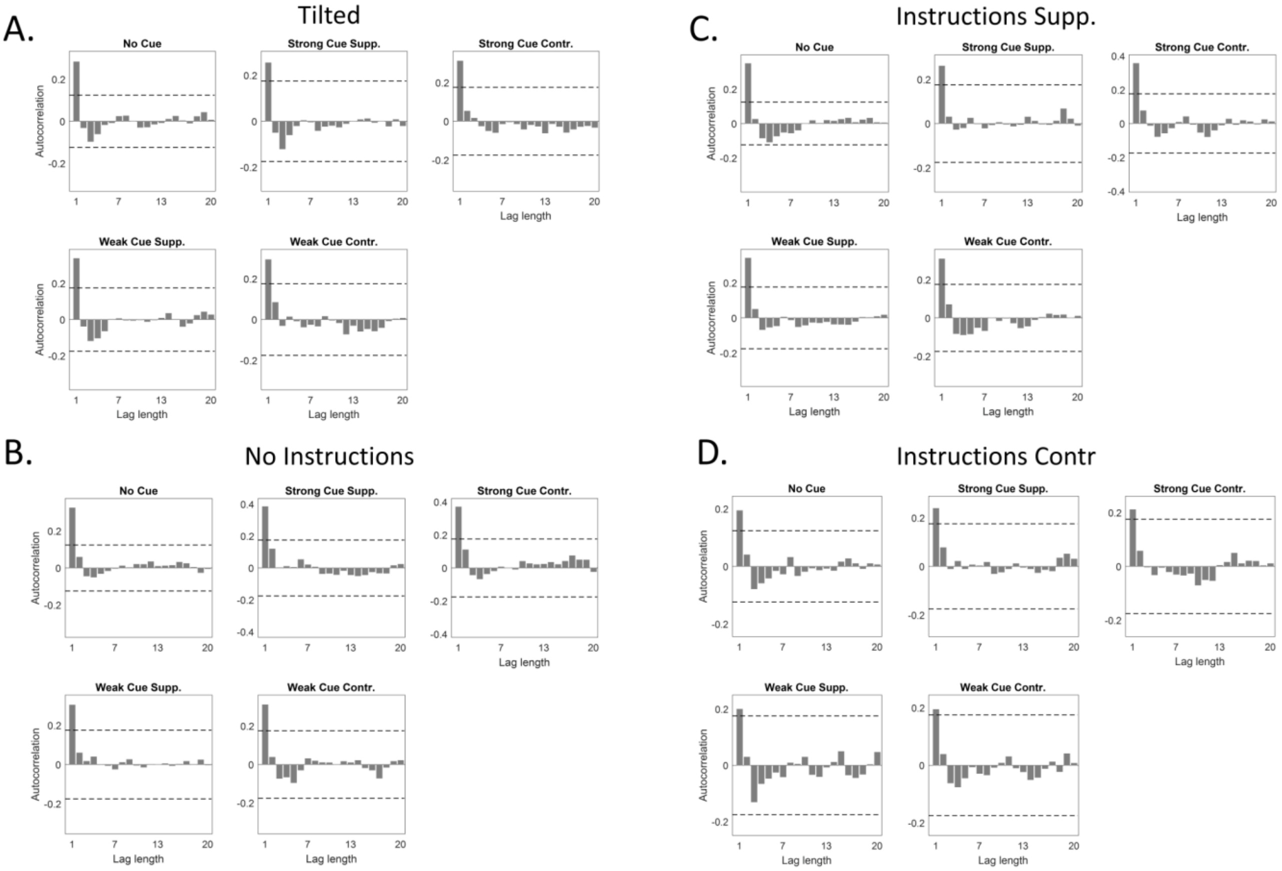


Dashed lines correspond to 95% confidence intervals for a white noise process. Interestingly, with the exception of small lags (lag = 1), no other point in any of the autocorrelation functions exceeds the dashed lines, meaning that the effects of history (at least for the average data) are restricted to small time differences and can be neglected. Consequently, none of our models took into account temporal statistics (see **Discussion** for further consideration of this issue).
