# Supplementary material for: Circular inference in bistable perception
Source: J Vis. 2020 Apr 21;20(4):12. doi: 10.1167/jov.20.4.12 (PMC7405786; doi:10.1167/jov.20.4.12)
Supplement: Supplement 9 [file jovi-20-4-12_s009.pdf]

Figure S9.
Transient preference (TP) as a function of time (black dots) and fitted power law model (blue curve).

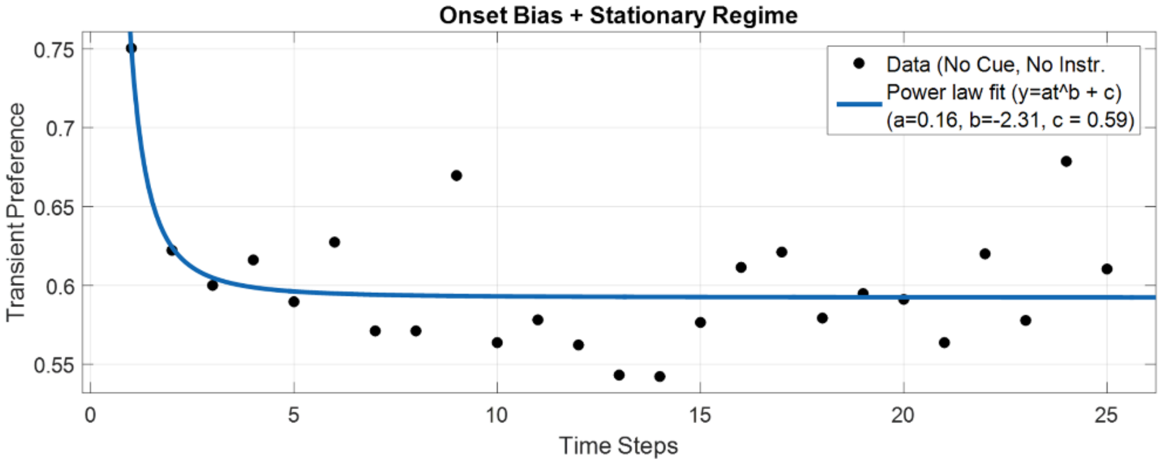

Transient preference can be defined as the RP for different time steps. As expected, TP attains a high value (TP(1) = 0.75) at the beginning of a run (t = 1), indicating the presence of an onset bias that rapidly decreases until reaching a stationary regime (TP(st. reg.) ~ 0.6) (Mamassian & Goutcher, 2005). Interestingly, TP in the stationary regime is above chance, indicating the presence of a persistent bias even after the onset bias fades out (the implicit bias that we describe in the **Main Text** is a combination of those two biases). This figure is reassuring, since absence of this pattern would indicate either a response bias or a very long inter-stimulus interval. This figure corresponds to the normal cube/no instructions/no cue condition (N=15), but a similar pattern was obtained for all other conditions as well (not presented).
