# Supplementary material for: Circular inference in bistable perception
Source: J Vis. 2020 Apr 21;20(4):12. doi: 10.1167/jov.20.4.12 (PMC7405786; doi:10.1167/jov.20.4.12)
Supplement: Supplement 10 [file jovi-20-4-12_s010.pdf]

Circular inference in bistable perception

Pantelis Leptourgos, Charles-Edouard Notredame,
Marion Eck, Renaud Jardri, Sophie Denève

Supplementary Information

Computational Modeling

The 3 models discussed in the **Main Text** (Naïve Bayes, Weighted Bayes and Circular Inference) were first introduced in this form in a previous study (Jardri, Duverne, Litvinova, & Denève, 2017). They assume that the brain is an "inference machine" whose goal is to combine new sensory information (sensory inputs) with accumulated knowledge from past experiences (priors) to make optimal predictions about the state of the world (Knill & Richards, 1996; Von Helmholtz, 1866) (optimality is not always achieved; see for example (Drugowitsch, Wyart, Devauchelle, & Koechlin, 2016) and the present CI model). Those inferences are largely based on internal, hierarchical representations of the causal structure of the world, which are called generative models (see for example **Figure 3A** in the **Main Text**). Generative models describe different hypotheses about the causes of the sensory input (bottom level). Higher levels correspond to more abstract and complex causes/variables.

The 3 models presented here describe different ways to implement hierarchical inference (or different ways to combine sensory inputs and priors). All are based on a powerful and general message-passing algorithm called **Belief Propagation** (BP; Bishop, 2006): Probabilistic messages are propagated locally between connected variables (nodes) in both directions, while posterior probabilities are computed by integrating all the available (at each level) information (the details are described in various textbooks). BP in a pairwise graph (all the variables have 1 parent at most) with binary variables is formalized using the following equations (see **Supplementary Materials** in (Jardri & Denève, 2013) for a detailed derivation):

$$L_i = \sum_j M_{j\to i} \qquad (S1)$$

$$M_{j\to i} = F\left(L_j - M_{i\to j}, w_{ji}^1, w_{ji}^0\right) \qquad (S2)$$

$M_{j\to i}$ is the probabilistic message from node j to node i (expressed as a log-ratio) and L_i is the belief (log-posterior ratio) about node (variable) i. $F()$ on the other hand corresponds to a sigmoid function that is defined as follows:

$$F(L, w^1, w^0) = log\left(\frac{w^1 e^L + w^0}{(1 - w^1)e^L + (1 - w^0)}\right) \qquad (S3)$$

w_{ji}^1 and w_{ji}^0 correspond to the strength of the $(j \rightarrow i)$ connection and are defined as the following conditional probabilities:

$$w_{ji}^1 = P\big(x_i = 1|x_j = 1\big), w_{ji}^0 = P\big(x_i = 1|x_j = 0\big) \qquad (S4)$$

and are equivalent to w_P (when j is above i) and w_S (when j is below i), which are used in the **Main Text**. Thus, this algorithm distinguishes between information (expressed in the form of the beliefs L) and the reliability of this information (the weights w). Strong evidence that is not trusted (as in the case of the disambiguated Necker cube; see also **Supplementary Figure S1B and C**) may exert a weaker effect on inferences compared to weak information that is highly trusted (e.g., a blurred image of a Necker cube in which some of the edges are missing and consequently it's compatible only with one interpretation).

NB and WB models are directly derived from equations *S*1 and *S*2, when assuming a generative model with 3 levels (variables), in which level 1 (bottom level) and level 3 (top level) correspond to observed variables (in our case, the visual cues and IB/Instructions, respectively) while level 2 (middle level) corresponds to a latent (unobserved) variable (the variable whose value we are trying to infer, namely, the interpretation of the cube). A graphical description of this generative model is presented in **Figure 3A**. Because top and bottom variables are observed, no messages are sent from the middle variable to them ($M_{2\rightarrow3} = M_{2\rightarrow1} = 0$). Consequently, the belief about the 3D interpretation is expressed in the following equation:

$$L_2 = M_{1\rightarrow2} + M_{3\rightarrow2} =$$

$$F(L_1, w_S) + F(L_3, w_P) = F(L_S, w_S) + F\big(L_{mpl} + L_{expl}, w_P\big) \qquad (S5)$$

where $w_P^1 = 1 - w_P^0$ and $w_S^1 = 1 - w_S^0$.

Equation *S*5 corresponds to the WB model and is based on the assumption that the brain uses probability matching to make decisions based on posterior probabilities ($L_{RP} = L_2$) (see the next section for a similar derivation of the more general case of a Softmax decision criterion).

In the NB model, the 2 weights are equal to 1. Thus, the information is perfectly trusted. Replacing w = 1 in equation *S*3, we obtain the following formula:

$$F(L, 1{,}0) = log(e^L) = L \qquad (S6)$$

Equation $S6$ shows that the model becomes linear in the case of very reliable information. By incorporating equation $S6$ into equation $S5$ we obtain the NB equation that was presented in the **Main Text:**

$$L_2 = L_S + L_{impl} + L_{expl} \qquad (S7)$$

Notably, equation $S7$ is the Bayes theorem expressed in log-ratios, indicating that when w = 1, the hierarchy is reduced to a single connection.

According to equation $S2$, messages (and consequently beliefs) depend not on beliefs of neighboring nodes per se, but on a rectified version of those beliefs (belief minus the message sent in the opposite direction). This rectification is crucial. Without it (or when it's partial), information has the tendency to be counted multiple times (**Figure 3B**), a form of suboptimal inference called **CI** (see (Deneve & Jardri, 2016; Jardri & Denève, 2013; Leptourgos, Denève, & Jardri, 2017) for more details). In CI, beliefs are computed using eq. $(S1)$, while messages adopt the following form (Jardri & Denève, 2013):

$$M_{j\to i} = F\left(L_j - aM_{i\to j}, w_{ji}^1, w_{ji}^0\right) \qquad (S8)$$

in which a has values ranging from 0 (no correction) to 1 (optimal inference, as in eq. $S2$).

In CI, beliefs (and messages) are calculated recursively (using eqs. $S1$ and $S8$), but these schemes are unable to be reduced to a simple equation, similar to eq. $S5$ (WB) or $S7$ (NB). For that reason, in our previous study, we considered an approximation of the CI algorithm, which is written as follows (Jardri et al., 2017):

$$\begin{aligned} L_2 = {} & F(L_1 + F(a_S L_1, w_S) + F(a_P L_3, w_P), w_S) \\ & + F(L_3 + F(a_S L_1, w_S) + F(a_P L_3, w_P), w_P) = S + P \end{aligned} \qquad (S9)$$

a_S and a_P represent the amount of overcounting of information (sensory inputs and priors, respectively) and can have any positive value (in the **Main Text**, the 2 terms are taken equal to 1). Equation $S9$ is structurally similar to eq. $S5$. The belief in level 2 is the sum of 2 non-linear terms, a sensory term (S) and a prior term (P). In contrast to eq. $S5$, both S and P also contain 2 additional terms, one that depends

on the belief of the level below (L_1) and another that depends on the belief of the level above (L_3). Those terms correspond to the reverberations (and reverberations of the reverberations) of information that result from the insufficient control of the propagated messages. Sensory inputs penetrate into and corrupt the feedback stream (the opposite for priors), resulting in aberrant correlations between sensory and prior effects (**Figure 3C**). Overall, eq. *S*9 maintains the main qualitative characteristics of CI (i.e., the amplification of information and the aberrant correlations between sensory inputs and priors), while it also contains a minimal number of free parameters.

Softmax Decision Criterion

In the case of the Softmax Decision Criterion, the probability of choosing the SFA (or SFB) interpretation is given by the following equations:

$$P(\hat{X} = SFA) = \frac{e^{\beta L_2}}{1 + e^{\beta L_2}} \qquad (S10)$$

$$P(\hat{X} = SFB) = \frac{1}{1 + e^{\beta L_2}} \qquad (S11)$$

where β is the temperature and controls the steepness of the curve ($\beta = 1$ corresponds to probability matching).

Because RP is by definition equal to $P(\hat{X} = SFA)$, we get:

$$L_{RP} = \log\left(\frac{RP}{1 - RP}\right) = \log\left(e^{\beta L_2}\right) = \beta L_2 \qquad (S12)$$

As a result, the 3 models (NB, WB and CI respectively) are written as follows:

$$L_{RP} = \beta\left(L_S + L_{impl} + L_{expl}\right) \qquad (S13)$$

$$L_{RP} = \beta\left(F(L_S, w_S) + F\left(L_{impl} + L_{expl}, w_P\right)\right) \qquad (S14)$$

$$L_{RP} = \beta\big(F(L_S + F(L_S, w_S) + F(L_{Pr}, w_P), w_S) + F(L_{Pr} + F(L_S, w_S) + F(L_{Pr}, w_P), w_P)\big) \qquad (S15)$$

Although a Softmax criterion can generate curves with slopes larger than 1, it cannot generate cue-prior interactions (see **Supplementary Figure S2**).

References Supplementary Information

Bishop, C. (2006). *Pattern Recognition and Machine Learning*. Springer.

Deneve, S., & Jardri, R. (2016). Circular inference: Mistaken belief, misplaced trust. *Current Opinion in Behavioral Sciences*, *11*, 40–48. https://doi.org/10.1016/j.cobeha.2016.04.001

Drugowitsch, J., Wyart, V., Devauchelle, A.-D., & Koechlin, E. (2016). Computational Precision of Mental Inference as Critical Source of Human Choice Suboptimality. *Neuron*, *92*, 1–14. https://doi.org/10.1016/j.neuron.2016.11.005

Jardri, R., & Denève, S. (2013). Circular inferences in schizophrenia. *Brain : A Journal of Neurology*, *136*(Pt 11), 3227–3241. https://doi.org/10.1093/brain/awt257

Jardri, R., Duverne, S., Litvinova, A. S., & Denève, S. (2017). Experimental evidence for circular inference in schizophrenia. *Nature Communications*, *8*, 14218. https://doi.org/10.1038/ncomms14218

Knill, D. C., & Richards, W. (1996). *Perception as bayesian inference*. New York, NY: Cambridge University Press.

Leptourgos, P., Denève, S., & Jardri, R. (2017). Can circular inference relate the neuropathological and behavioral aspects of schizophrenia? *Current Opinion in Neurobiology*, *46*, 154–161. https://doi.org/10.1016/j.conb.2017.08.012

Mamassian, P., & Goutcher, R. (2005). Temporal dynamics in bistable perception. *Journal of Vision*, *5*(4), 361–375. https://doi.org/10:1167/5.4.7

Moreno-Bote, R., Knill, D. C., & Pouget, A. (2011). Bayesian sampling in visual perception. *Proc Natl Acad Sci U S A*, *108*(30), 12491–12496. https://doi.org/10.1073/pnas.1101430108

Von Helmholtz, H. (1866). Concerning the perceptions in general. *Treatise on Physiological Optics Iii*.
